# Multiparameter laser performance characterization of liquid crystals for polarization control devices in the nanosecond regime

**DOI:** 10.1038/s41598-022-14974-5

**Published:** 2022-06-29

**Authors:** Kenneth L. Marshall, Kyle R. P. Kafka, Nathaniel D. Urban, Jason U. Wallace, Stavros G. Demos

**Affiliations:** 1 Laboratory for Laser Energetics, University of Rochester, Rochester, NY USA; 2 Department of Chemistry, D’Youville College, Buffalo, NY USA

**Keywords:** Materials for optics, Materials chemistry, Organic chemistry, Photochemistry, Optical materials and structures

## Abstract

Interactions of liquid crystals (LC’s) with polarized light have been studied widely and have spawned numerous device applications, including the fabrication of optical elements for high-power and large-aperture laser systems. Currently, little is known about both the effect of incident polarization state on laser-induced–damage threshold (LIDT) and laser-induced functional threshold (LIFT) behavior at sub-LIDT fluences under multipulse irradiation conditions. This work reports on the first study of the nanosecond-pulsed LIDT’s dependence on incident polarization for several optical devices employing oriented nematic and chiral-nematic LC’s oriented by surface alignment layers. Accelerated lifetime testing was also performed to characterize the ability of these devices to maintain their functional performance under multipulse irradiation as a function of the laser fluence at both 1053 nm and 351 nm. Results show that the LIDT varies as a function of input polarization by 30–80% within the same device, while the multipulse LIFT (which can differ from the nominal LIDT) depends on irradiation conditions such as laser fluence and wavelength.

## Introduction

For over 70 years, polarized light interactions with liquid crystals (LC’s) have been widely studied and have given rise to numerous optical and laser device applications (e.g., circular polarizers, wave plates, beam shapers, vortex polarizers) ranging from the near-ultraviolet (UV) to radio-frequency wavelengths^1–14^. Both active and passive LC technologies and materials have proven themselves useful as robust and cost-effective components for demanding applications in high-peak-power laser systems^1,2,6,7,11,12,15–17^. Passive LC circular polarizers and wave plates have been employed on the 60-beam, 60-TW OMEGA Nd:glass laser system at the University of Rochester’s Laboratory for Laser Energetics for over 30 years in the high-peak-power near-infrared (IR) sections of the OMEGA laser driver line^1,11^ as well as in state-of-the-art petawatt-class laser systems^12^. Their numerous advantages include scalability to large apertures, cost effectiveness, high optical quality and contrast, broad angular tolerance, and laser-induced–damage thresholds (LIDT’s) for optimized materials at 1054 nm of > 30 J/cm^2^, 3 J/cm^2^, and 1 J/cm^2^ at 1-ns, 10-ps, and 600-fs pulse durations, respectively^17^. These advantages are largely driven by the anisotropy inherent to LC materials; for low-molar-mass nematic and chiral-nematic LC’s, their fluidity enables large-aperture device scalability, while their optical anisotropy, or birefringence (commonly expressed as Δ*n*), provides polarization-dependent optical properties.

**Figure 1:**
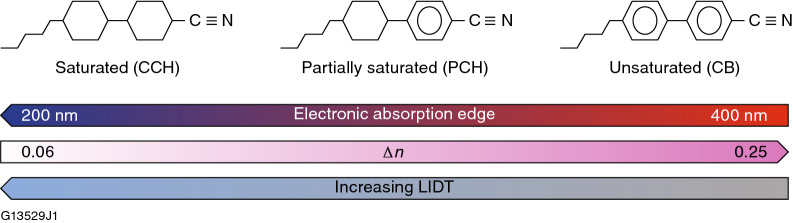
Molecular structures for the three classes of LC compounds in Table 1 and a graphical representation of dependence of optical absorbance, birefringence, and laser damage with respect to these classes.

The optical absorption spectrum, birefringence, and LIDT of LC materials are all influenced strongly by their molecular structure. In general, reducing the number of double bonds in the molecule by increasing its degree of “saturation” (which eliminates delocalization of π-electron density and polarizability) shifts the optical absorption edge to shorter wavelengths and improves the LIDT at the cost of a reduction in LC birefringence (Fig. 1)^15–17^. The anisotropic ordering arises from both molecular shape and electrostatic van der Waals interactions between functional groups on neighboring molecules, where the net average, long-range orientation of the long molecular axes is defined as the director $\left( {\hat{n}} \right)$ and quantified by the order parameter *S*, defined as $S = \left( {{1 \mathord{\left/ {\vphantom {1 2}} \right. \kern-\nulldelimiterspace} 2}} \right)\left\langle {3\cos^{2} \theta - 1} \right\rangle ,$ where θ is the angle between the director and the long axis of each molecule^18^. The two most-common LC materials classes used in laser applications are the nematic and chiral-nematic (cholesteric) phases. The director in the nematic phase is unidirectional, whereas in the chiral-nematic phase, the chiral group on the molecule imparts a helical twist sense to the LC phase with a spatial periodicity defined as its pitch length (*p*).

A primary requirement in nearly all LC optical devices (and especially for laser applications) is that the LC material be constrained to adopt a high degree of homogeneous and monodomain molecular orientation over the device’s clear aperture. Numerous boundary surface treatments exist for reinforcing monodomain LC orientation, the most commonly used of which are buffed polymer alignment layers. Because surface-anchoring techniques have the potential to affect the laser-induced–damage behavior, LIDT evaluation has historically been performed on LC materials in long fluid path lengths (50 to 100 *µ*m) constructed without alignment layers. Such testing was conducted in order to gain an understanding of the LC material’s behavior under exposure to high-energy laser pulses isolated from potential effects induced by physicochemical interactions with surface-anchoring layers and conditions (LC elastic constants, boundary molecular tilt angle, alignment materials chemistry and application methods)^15–17^. In such devices where extrinsic forces (e.g., electric fields, alignment layers) to enforce uniform LC director orientation are absent, the resultant optical output depends primarily on the LC material’s order parameter *S*, and the incident laser polarization encounters a multidomain structure with the output representing an averaging of the ordinary (*n*_o_) and extraordinary (*n*_e_) refractive indices of the LC molecules. For LC materials with high birefringence (Δ*n* ≥ 0.1), it becomes necessary to conduct testing at an elevated temperature above the material’s LC-to-isotropic phase transition in order to visualize damage that would otherwise be obscured by forward-scattered light from the multidomain LC structure. This method is useful for general screening of LC materials by chemical class to determine laser survivability and for certain applications where a highly uniform, monodomain orientation of the LC material at the substrates is not essential (e.g., LC circular polarizers). One shortcoming of such long-path-length testing is that it gives very little insight on how the LC’s LIDT may be affected in device applications where the LC molecules are constrained in a monodomain alignment state induced by contact with substrates bearing a polymer alignment layer (e.g., wave plates, mirrors, and beam shapers). In such cases, the alignment state may produce variations in optical behavior as a function of laser beam polarization that may arise through various mechanisms (e.g., molecular orientation, chemical interactions, or generation of electric-field enhancements within the material). The experiments described in this work were designed to test the performance limits of such highly oriented LC devices under intense pulsed-laser irradiation with various input polarization states to evaluate not only the classical localized LIDT of the LC material, but also its *functional performance* with respect to accumulated doses of laser pulses over a range of applied fluence.

Because of their fluid nature, laser-induced damage in LC’s is evidenced first by the formation of laser-induced cavitation bubbles, followed by carbonaceous residues and substrate damage at sufficiently high laser fluences^1,11,15–17^. Such gas bubbles formed as the result of nano- to microscale material decomposition can exhibit *self-healing* behavior by reabsorption into the surrounding LC fluid in seconds, minutes, or longer (depending on the size of the bubbles and their diffusion rate through the unaffected LC fluid). Optical detection of transient bubble formation and reabsorption by the dark-field microscopy technique used commonly for LIDT testing can be useful as a general fingerprint indicator of damage onset, but it reveals little if any information on laser-induced localized changes in molecular alignment that could have a detrimental effect on the functional performance of an LC device. This issue becomes especially important for LC polarization control device applications since the relationship between laser input polarization and laser-induced–damage and functional performance in LC devices remains largely uninvestigated.

The goals of this work are to expand the characterization of the performance of LC devices for laser applications using two previously unexplored methods: (1) characterization of the LIDT dependence on the incident-light polarization state by examining the influence of the oriented LC on its laser-induced breakdown and (2) evaluation of the conditions (e.g., laser fluence, pulse duration, wavelength, and number of pulses) under which the LC molecular order is modified or degraded by sufficient incident laser energy, as evidenced by the deviation of the transmitted polarization state from the device’s design parameters. To achieve these goals, the standard damage-testing protocol for LC materials based on ISO21254-1: 2011 (Lasers and laser-related equipment -Test methods for laser-induced–damage threshold)^19^ and implemented as described in Ref.^17^ was expanded to explore a “laser-induced functional threshold” (LIFT), defined as a reduction in one or more system-defined, key device functional parameters (e.g., transmission, reflection, birefringence, polarization rotation, contrast) that may occur at fluences lower than those required to produce the visible and permanent evidence of material modification typically defined as laser-induced damage. The point at which the value of LIFT drops below a system-defined tolerance metric is taken as the LIFT “trigger point,” which for the purposes of this study was defined as a reduction in transmission to < 98%. To this end, a novel detection system was developed that is capable of detecting the onset of both performance degradation (LIFT) and classical laser damage (LIDT). This new test bed was used to study LIDT and LIFT characteristics of several LC mixture compositions in both circular polarizers and wave-plate device geometries. For LC circular polarizer devices, the LIDT varied as a function of incident circular polarization handedness by a factor of 30% to 80% for a given sample. To our knowledge, such angular dependence of high-peak-power LIDT on incident polarization in LC materials has not been reported previously. The results suggest that multipulse functionality was best preserved in LC devices having the highest degree of saturation.

## Experimental

### LC mixtures

A series of four different LC mixtures prepared using both commercially available LC compounds and those synthesized in-house were employed in this study. Relevant material properties are summarized in Table 1; generic molecular structures for each material class in Table 1 are shown in Fig. 1. These compounds varied both in their degree of saturation and helical pitch length.

**Table 1:** Composition, properties and applications of LC mixtures used in the LIDT studies.

LC mixture	Phase type	Material class	Chiral components (wt%)	Absorption edge (nm)	Director twist angle (10-*µ*m cell)	Application
NU131-LCP	Chiral nematic	CCH/CB	19.4	355	~ 3600° (* λ*_r_ = 1053 nm)	Circular polarizer/isolator
6601-1CB			1	315	~ 270	Polarization rotator
N1646-WP	Nematic	PCH/CCH	0	324	0	Wave plate
LLE1202		CCH		294		

CB-15 (EM Chemicals) was used as the chiral dopant in the chiral-nematic mixtures.

*CCH* Cyclocyclohexane, *PCH* Phenylcyclohexane, *CB* Cyanobiphenyl.

**Figure 2:**
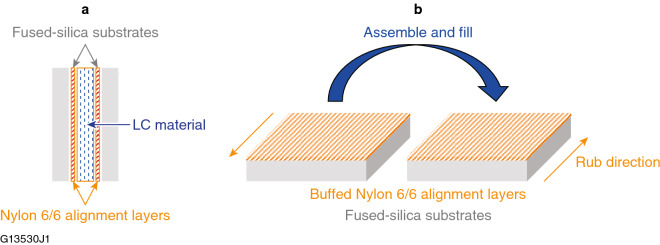
(**a**) Side view of a 10–12-*µ*m-thick LC device prepared using fused-silica substrates bearing spin-deposited nylon 6/6 coatings on their surfaces for LIDT/LIFT testing. The fluid gap thickness is determined by spacer beads embedded in the epoxy resin used to bond the substrates to each other. (**b**) For LC wave-plate devices, the nylon 6/6 layers were buffed in opposite directions (antiparallel) prior to assembly to minimize the effect of LC molecular pre-tilt at the surfaces and achieve monodomain orientation.

Figure 2 shows a schematic diagram of the test cells used for LIDT testing under conditions where both the LC molecular orientation and the incident polarization state are well defined. Methods for preparing well-aligned LC devices for laser applications have been documented extensively in the literature^1,5,6,11,18–20^. In brief, highly oriented LC devices for laser-damage testing were assembled in class-100 clean room conditions using ultrasonically cleaned 2-in.-diam fused-silica substrates that have a nylon 6/6 coating spin-deposited on the inner-facing surfaces of the assembled device. The LC fluid gap is controlled using spacer beads (10 to 12 *µ*m) mixed in an epoxy adhesive that is also used to affix the substrates together. The cell gap is filled with the LC fluid by capillary action. When the selected LC fluid is a chiral-nematic composition, the LC device functions as a wavelength-specific circular polarizer and is assembled without surface orientation to avoid specular reflection from the chiral-nematic helical structure. For wave-plate devices, monodirectional LC orientation was generated by buffing the nylon 6/6 coatings using a commercial buffing machine prior to device assembly.

### LIDT and LIFT testing

**Figure 3:**
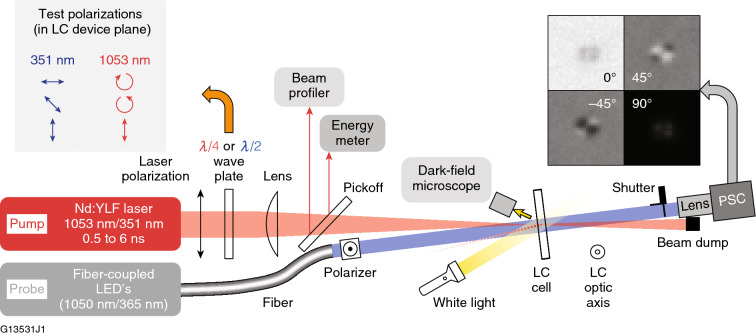
Schematic diagram of the nanosecond laser system used to conduct laser damage testing in oriented LC devices (PSC = polarization-sensitive camera). The optic axis orientation of the LC cell is in the plane of the substrate surfaces and orthogonal to the plane of the page.

The laser source employed in these experiments was a nanosecond laser system operated at either its fundamental wavelength (1053 nm) or the third harmonic (351 nm). Temporal profile shaping of the seed laser is accomplished using an arbitrary waveform generator that yields amplified pulses ranging from 0.5-ns to 6-ns duration with 0.1-ns resolution. A schematic of the damage-testing system is shown in Fig. 3.

Beam profiling and pulse energy diagnostics allowed precise determination of the peak laser fluence for each incident pulse with a Gaussian beam diameter of either ~ 350 *µ*m (351 nm) or 450 *µ*m (1053 nm). The polarization state of the laser beam was controlled using suitable wave plates. Dark-field microscopic sample observation with white-light illumination was used to detect *in-situ* laser damage for LIDT measurements. This conventional approach is widely used for damage detection in solid-state optics (e.g., coatings, mirrors, and substrates). The onset of laser damage was visualized by a detectable change in the localized scattering intensity in the irradiated region determined by subtraction of images recorded prior to and immediately after laser irradiation. The LIDT measurements reported in this work are expressed in terms of damage probability using the “limit cumulative” method^21–23^, which is designed to increase the statistical confidence when extensive testing is not possible. In the present study, we considered that damage testing could cause molecular decomposition due to ablation within the LC fluid that can result in an artificial lowering of the observed LIDT or LIFT values. The limit cumulative method aids in mitigating this risk by calling for a total of ≥ 30 test sites irradiated at various fluences. The reported LIDT for each sample is defined as the lowest fluence at which damage was observed, which corresponded to a damage probability of approximately 5–10%.

For LIFT measurements, the standard LIDT test bench was augmented using an in-house–developed polarimetry imaging system consisting of (a) fiber-coupled polarized LED light sources to produce a collimated “probe” light beam that traverses the area of the sample exposed to the laser beam, (b) a suitable combination of a polarizer and wave plate located in front of the fiber output collimation lens to control the polarization state of the probe light, and (c) and a polarization-sensitive monochrome camera capable of distinguishing between four linear polarization states simultaneously using on-sensor wire-grid polarizers (Thorlabs CS505MUP). The wavelength of the polarized LED probe beam was chosen to match the damage test laser’s pump-beam wavelength, which is also the LC device-specific operational wavelength. The LIFT was determined by performing an accelerated lifetime test on the LC device that is defined by the number of pump-beam pulses delivered to the sample at a given fluence until functional damage was observed. The polarization-sensitive detection system monitors the change of transmission and polarization state of the probe LED beam as it passes through the LC device following exposure to the pump-beam laser pulses. A change in either transmission or polarization state detected during multipulse laser exposure that exceeds a predetermined device specification is defined as the LIFT for that particular device.

An acceptable value of LIFT will depend on both the LC device application and the performance requirements of the optical system in which the LC device is used, including the laser fluence, wavelength, duration and number of exposure pulses. For the purposes of this study, we defined an acceptable LIFT survivability to be a polarized light transmission of ≥ 98%. Testing using lower transmission threshold values yielded very similar results because the degradation process that occurs when the sample's transmission is below 98% is rather rapid. For example, re-analysis of the current data using an adjusted transmission threshold of < 90% resulted in a LIFT value increase of no more than 10%.

The chiral-nematic mixtures NU131-LCP and 6601-1CB (Table 1) were evaluated in a circular polarizer geometry at 1053 nm using three different polarization input states (left-circular, right-circular, and linear) with the LC mixture’s helical pitch adjusted by composition to produce transmitted circularly polarized light (and selective reflection) at 1053 nm. A *λ*/4 wave plate was used to convert the input linear polarization state from the pump-laser beam into right-circular or left-circular polarization. Because the chiral nematic LC mixtures evaluated employed CB15 as the chiral dopant (right-handed twist), the resultant mixtures with nematic hosts transmit right-handed circularly polarized light. Nematic LC mixtures N1646-WP and LLE1202 were evaluated at 351 nm in test devices with antiparallel alignment generated by unidirectional buffing of the nylon coating on each of the substrates (wave-plate geometry). For these experiments, the LIDT was measured using a 351-nm *λ*/2 wave plate to generate incident linear polarization oriented at 0°, 45°, or 90° to the LC director. The LC fluid layer thickness for all devices was 10 to 12 *µ*m. All samples were mounted in the laser-damage test setup with the input surface of the device set at an incidence angle of 7° with respect to the pump-laser beam.

## Results and discussion

### 1053-nm circular polarizer/selective reflector using NU131-LCP

**Figure 4:**
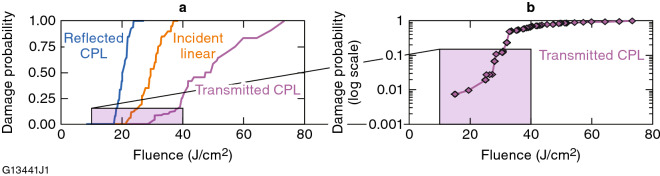
(**a**) Damage probabilities for the chiral-nematic LC device containing NU131-LCP as a function of 1053-nm, 1.4-ns laser fluence and incident polarization. (**b**) Damage probabilities for transmitted CPL pulses incident on the device at low fluence [corresponding to the inset in Fig. 5(a)]. These damage probability data are plotted on a logarithmic scale and represent an additional 250 sites of 1-on-1 damage data collected by line-scanning the sample.

**Figure 5:**
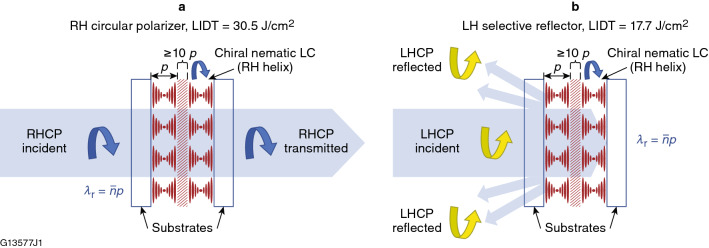
Propagation of right-handed circularly polarized (RHCP) and left-handed circularly polarized (LHCP) light through an LC device containing a chiral nematic LC doped with CB-15 as the chiral twisting agent. (**a**) incident circular polarized light with the same twist sense as the LC helix (RH) is transmitted, whereas in (**b**) for the same device, incident circular polarization of the opposite handedness (LH) is selectively reflected due to Bragg scattering. A cell thickness of at least ten pitch lengths (*p*), indicated by the area filled with diagonal slashes near the center of the cell, is required to observe these effects with sufficient magnitude for device applications.

Figure 4 shows the single-pulse damage probability for the NU131-LCP circular polarizer sample for distinct input polarization states. Right-hand circularly polarized (RHCP) and left-hand circularly polarized (LHCP) light are defined following the convention used in the optics and physics community^24^, in which the electric field vector rotates either clockwise or counterclockwise, respectively, from the point of view of the receiver (i.e., viewing into the light source and opposite to the direction of light propagation^25^. Figure 5 shows the optical behavior for the same device depending on whether the input beam polarization has the twist sense that corresponds to the device application as either a circular polarizer or a selective reflector. The highest LIDT (30 J/cm^2^) was observed when the handedness of the incident circular polarization matched the helical twist direction of the LC device, which corresponds to a highly transmissive state [transmitted circularly polarized light (CPL) in Figs. 4a and 5a]. Conversely, the lowest LIDT (17 J/cm^2^) was observed for incident circular polarization of the opposite handedness to that of the LC device [reflected CPL, Figs. 4a and 5b], which corresponds to Bragg-like selective reflection for a bandwidth of light defined by *λ*_r_ = *np*, where *λ*_r_ is the selective reflection wavelength, *n* is the average refractive index of the LC material, and *p* is the helical pitch length^1,2,11^. For incident linear polarization, the LIDT was nearly midway between the LIDT values for incident left-circular and right-circular polarizations, which is consistent with the representation of linear polarization as an equal combination of right- and left-circularly polarized states.

The dependence of LIDT on the incident polarization state may be explained by the difference in electric-field enhancement within the chiral-nematic LC helical structure. Laser damage is governed by the local fluence and electric field intensity. The overlap within the LC material of the incoming and reflected beams increases the local fluence/intensity to a value correspondingly higher than the nominal value of the input beam. This effect in turn causes a reduction of the damage threshold compared to that induced only by the input beam (i.e., local excitation conditions). This local electric-field enhancement and its effect on LIDT is well established in conventional solid-state optics (thin-film coatings, surface structures, the exit surfaces of transparent optics, or from particulate contamination). Interaction of incident polarization of the opposite handedness to that of the LC circular polarizer produces electric-field enhancement due to the Bragg-like selective reflection introduced by the counter-rotating polarization components^26,27^ and could explain the difference in the measured damage thresholds. This interaction of incident polarization and LC chirality may also imply that localized alignment defects in the LC material could become the source of site-specific electric-field enhancement and initiate the formation of laser-induced‒damage precursors in LC circular polarizers. These devices are constructed intentionally to contain a certain amount of *focal conic defects* (areas of antagonistic anchoring where the molecular ordering bends around a circular orientation on one surface and follows a straight line running from the circle center to the opposing surface) to eliminate backscattered specular reflection, which could damage laser system components^1,11^.

An additional 250 sites of 1-on-1 (one test pulse per site) LIDT data at 1053 nm, 1.4 ns were measured on the circular polarizer device containing NU131-LCP to assess the damage probability for transmitted CPL at lower fluences Fig. 4b. A very small number of damage sites were still detected at the ~ 1% probability level at ~ 15 J/cm^2^, which is near to the LIDT value for reflected CPL (17.1 J/cm^2^). These results potentially suggest that this type of isolated damage may be due to highly localized inversion of chirality (twist sense) of the LC material produced by photochemically induced molecular rearrangement and/or interactions with alignment coating defects. Chirality inversion will initiate backscattering through the formation of focal conic defects and therefore increases the local fluence/intensity inside the LC along the beam path as the beam impinges on the coating defect. Such an event will locally reduce the damage threshold, which manifests as isolated locations of reduced LIDT.

Both LIDT and LIFT testing were conducted at 1053 nm on the same sample in areas of the device that had not yet been subjected to incident laser energy. No significant differences between the LIDT and LIFT fluence values were observed for this sample at 1053 nm.

### LIDT testing

**Figure 6:**
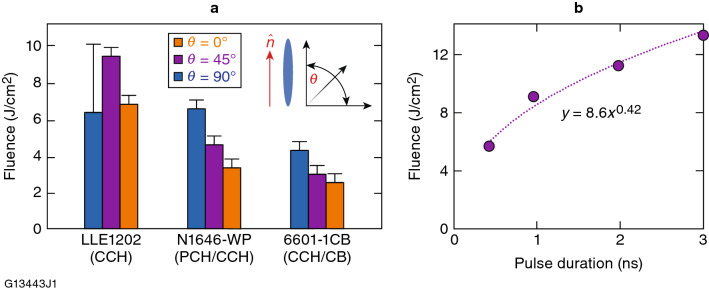
The 1-on-1 LIDT results at 351 nm. (**a**) LIDT at 1-ns duration as a function of incident linear polarization angle *θ* with respect to the LC director ñ. Uncertainty bars extend to the nominal 0% and 100% damage probability fluences. (**b**) 1-on-1 LIDT for LLE-1202 as a function of pulse duration with *θ*  = 45°.

The LC materials compositions 6601-1CB, N1646-WP, and LLE 1202 (Table 1) were tested at 351 nm in devices bearing buffed nylon 6/6 layers on their inner surfaces and assembled so that the rub directions were antiparallel, as shown in Fig. 2b (i.e., the standard cell geometry used for LC wave plates). No attempt was made to adjust the LC fluid layer thickness or its birefringence to optimize device retardance at 351 nm. Figure 6a depicts the 1-on-1 LIDT test results for these three LC compositions for incident laser polarization angles *θ* of 0°, 45°, and 90° to the LC director in each device [shown as an inset in Fig. 6a]. The 1-on-1 LIDT values for each incident polarization state are portrayed as color-coded columns that represent the lowest fluence at which damage was observed within the statistical sample size of the damage test. In general, all three materials follow the trend of increasing LIDT as the angle between the incident polarization and the LC director approaches 90°, with higher LIDT values attained by those materials that possess a higher degree of saturation [CCH > PCH/CCH > CCH/CB, Fig. 3a]. To our knowledge, such angular dependence of high-peak-power LIDT on incident polarization in LC materials has not been reported previously.

The measured 1-on-1 LIDT values of these three LC materials is a direct function of the electronic structure of each mixture’s individual components^17^. The chiral-nematic mixture 6601-1CB contains a significant amount of unsaturated CB-15, while LLE1202 contains only completely saturated components, which results in an absorption spectrum that is blue shifted toward 200 nm. LLE1202, the most highly saturated mixture evaluated, showed the highest 1-on-1 LIDT values in this group, with a maximum value of 9 J/cm^2^ for incident polarization aligned 45° to the LC director. This result represents the optimal geometry for employing this material in an LC wave plate since the linear birefringence of the device in this orientation is maximized. Although the data set for LLE1202 showed a lower-than-expected LIDT for *θ*  = 90°, the upper uncertainty bar remains consistent with the trend of progressively increasing 1-on-1 LIDT values as *θ *approaches 90°, as observed in both 6601-1CB and N1646-WP. The LIDT value at the lower uncertainty bar is at nearly the same level as the LIDT for *θ*  = 0°. We hypothesize that the dependence of the LIDT on the input polarization arises from the correlation of the absorption cross section as a function of the LC director orientation. Samples 6601-1CB and N1646-WP may exhibit higher absorption when the electric field is polarized parallel to their long molecular axes. The underlying cause of the observed small deviation of the behavior of LLE1202, with respect to its LIDT at *θ* *θ* = 90°, remains to be investigated. We hypothesize that for this particular LC material composition the deviation may be related to changes in LC molecular orientation on the alignment layers as has been reported under both cw UV^28–30^ and pulsed-laser exposure conditions^31^.

The 1-on-1 LIDT behavior for LLE1202 was also evaluated at different pulse lengths (0.5 to 3 ns) with the device in the wave-plate orientation (*θ*  = 45°); the results are shown in Fig. 6b. A power-law fit as a function of the pulse duration to the data yields an exponent value of approximately 0.42.

### LIFT testing

**Figure 7:**
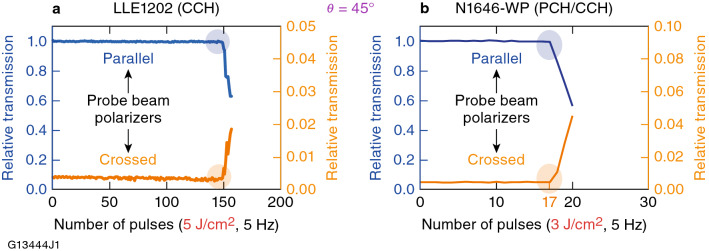
Multipulse LIFT damage testing data for (**a**) LLE1202 and (**b**) N1646-WP at 351 nm, 1 ns with a 5-Hz pulse repetition rate. The blue- and orange-shaded circles indicate the LIFT for each material composition and testing conditions.

Functional testing was performed on the antiparallel-aligned 6601-1CB, N1646-WP, and LLE1202 to quantify the performance limits of these LC materials under multiple pulse exposure conditions. The incident laser polarization angle *θ* with respect to the LC director was maintained at 45°, which is the typical orientation at which the device would be used in a wave-plate application. The LIFT pulse fluence used for each LC material was determined by evaluating the extensive LIDT data of the form described in Sect. 3.2*.* The pulse repetition rate used for the LIFT measurements was 5 Hz*.* Figure 7 shows the output from the polarization-sensitive imaging system (Fig. 3) while monitoring the parallel and orthogonal polarization components of the transmitted linearly polarized probe LED light beam through the laser-irradiated sites in devices containing LLE1202 and N1646-WP. Figure 7a shows transmission data through LLE1202 for both probe-beam polarizations (maximum when the probe-beam polarizers are parallel and minimum when the probe-beam polarizers are orthogonal) as a function of the number of 351-nm laser pulses delivered to the sample at 5 J/cm^2^ and 5-Hz repetition rate. The LIFT in this case occurs at ~ 150 pulses, the point at which both the probe-beam transmission is reduced through parallel polarizers and is increased through orthogonal polarizers. This behavior could be due to either a reduction in the LC optical anisotropy due to photolytic decomposition or a change in the in-plane surface orientation or pretilt angle of the LC material. Examination of Fig. 7a shows that there is an imbalance in the total signal decrease with parallel probe-beam polarizers and signal increase with orthogonal probe-beam polarizers, as evidenced by the different vertical axis scales for the two polarizations. This finding suggests that significant probe beam energy is being lost to absorption and/or scattering in the area of the material that is exposed to the 5-J/cm^2^ pump fluence. Additional experiments conducted with a range of laser fluences show that the amount of energy loss (reduced parallel minus increased orthogonal) varied as a function of fluence, where lower laser fluences produced less energy loss due to scattering.

The LIFT cannot be assigned to thermal effects due to the very low absorption by the material and the relatively low 5-Hz repetition rate of the test laser system. Changes in temperature would have also been detected with the polarimeter detection system. On specific sites, the LIFT appears to be permanent for exposures at lower fluences and higher number of pulses, which suggests that there is some level of damage occurring in the alignment layer. For the opposite case of higher fluences and lower number of pulses, the polarimeter system observes recovery between pump pulses, but subsequent pump pulses create a larger effect. This behavior appears to be due to a combination of LC material and alignment layer failure.

Multipulse LIFT data collected on N1646-WP [a mixture of partially saturated (PCH) and fully saturated (CCH) materials (Table 1 and Fig. 1)], tested under similar conditions as for LLE1202, exhibited a lower overall functional fluence threshold. The results shown in Fig. 7b, obtained at a lower fluence (3 J/cm^2^), indicate this material undergoes a loss in functionality after only 17 pulses at 5 Hz, as evidenced by measurable changes in its probe-beam transmission. Due to its fully saturated LC composition, LLE1202 outperforms N1646-WP significantly, as evidenced by its ability to withstand exposure to over 400 pulses at the same fluence (3 J/cm^2^ and 5-Hz repetition rate) before changes in transmitted polarization intensity are noted.

**Figure 8:**
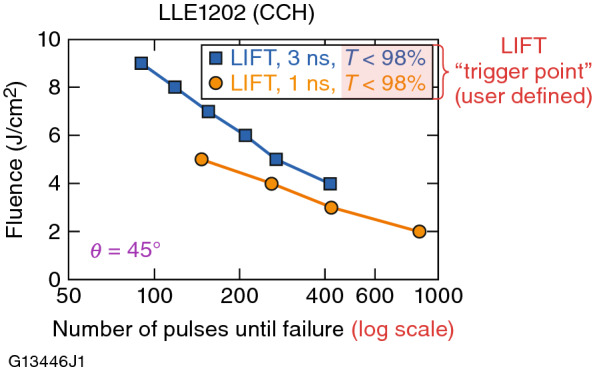
LIFT results for LLE1210 at 351 nm for both 1-ns and 3-ns laser pulses delivered at a 5-Hz repetition rate. The high saturation of this CCH-based LC material allows it to withstand nearly 1000 pulses at 2 J/cm^2^.

The LIFT performance of the fully saturated LC mixture LLE1202 was characterized further at 351 nm by evaluating its multipulse performance as a function of both fluence *and* pulse duration (Fig. 8). Each data point in Fig. 8 corresponds to the number of pulses delivered to the sample at a given wavelength fluence and pulse width before the polarized light transmission *T* drops below a chosen LIFT “trigger point” (for this work, *T* < 98% *T*_0_, or a 2% reduction in transmission). These results demonstrate that the 351-nm LIFT for LLE1202 depends strongly on the laser fluence, as well as the pulse duration and number of exposure pulses. The dependence of the maximum number of pulses on the pulse duration (e.g., at an fluence of 5 J/cm^2^, 150 and 270 pulses at 1 ns and 3 ns, respectively, are required to reach the LIFT) indicates that the functional threshold degradation is not governed by a purely linear process.

**Figure 9:**
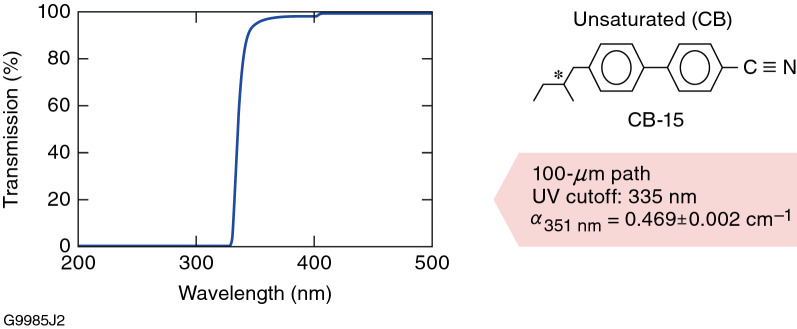
Molecular structure and optical absorbance data for a 100-*µ*m pathlength of pure chiral-dopant CB-15 used in 6601-1CB at a 1-wt% doping level.

The influence of a low concentration of a UV-absorbing material on the multipulse LIFT behavior of a saturated LC host material was studied in 6601-1CB, a saturated nematic LC, containing ~ 1 wt% of the unsaturated chiral dopant CB-15 (Table 1 and Fig. 1). This chiral dopant is used widely in many LC device applications by virtue of its high helical twisting power. The molecular structure and absorption spectrum of CB-15 are shown in Fig. 9; because its UV absorption cutoff occurs around 355 nm, it has only limited usefulness in high-peak-power UV laser applications^5,15,20^.

**Figure 10:**
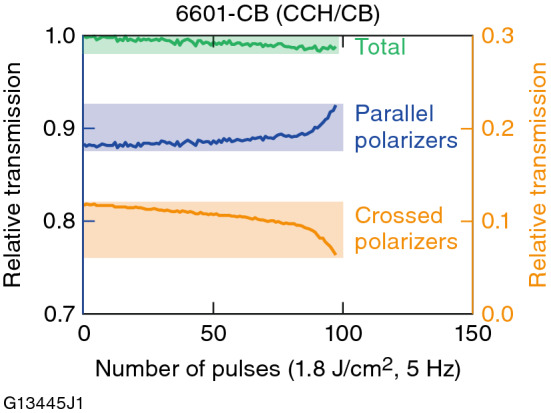
Multipulse LIFT testing at 351 nm for 6601-CB. Addition of unsaturated chiral-dopant CB-15 in concentrations as low as 1 wt% produces nearly immediate degradation under multipulse exposure.

Figure 10 shows the multipulse LIDT behavior of the 6601-1CB chiral-doped mixture at 351 nm upon irradiation with 1.8-J/cm^2^, 1-ns pulses at 5 Hz. Due to the circular birefringence imparted to the LC mixture by CB-15, the initial transmission values were not 1 and 0 for parallel and crossed probe-beam polarizers, respectively.

Contrary to what was observed in both LLE1202 and N1646-WP, the corresponding transmission curves as a function of the number of exposure pulses for 6601-1CB show measurable functional changes manifested as a nearly immediate drift in transmission after irradiation, with a greater functional change onset near ~ 85 pulses at 1.8 J/cm^2^. The sum of the transmission values from the parallel and crossed probe-beam polarizations (green curve in Fig. 10) also shows a steady decline, indicating an increasing amount of optical loss due to scattering and/or absorption as the number of laser pulses increases. The immediate onset of degradation along with the continuing change in transmission for both polarizations that increases with laser exposure is strong evidence that CB-15 is being consumed by a photolytic process, with a concomitant increase in the mixture’s helical pitch length. Similar photoinduced changes in LC properties (birefringence, viscosity, and electro-optic response) in cyanobiphenyl-based LC’s and other LC compounds and mixtures are known to occur and have been reported previously^32–36^.

## Conclusions

The multipulse laser-damage behavior of a series of LC host mixtures containing components with differing degrees of saturation was determined in practical device geometries in which the LC director was constrained to adopt a monodomain or nearly monodomain orientation. In testing at 1053 nm and 351 nm, the LIDT behavior in these materials depends significantly on the incident polarization state for laser light encountering the input surface of the LC test device at near-normal incidence (7°).

Certain compositions of saturated, UV transparent nematic LC mixtures evaluated in a wave-plate geometry displayed remarkable robustness in LIFT testing at 351 nm, with one LC mixture (LLE1202) being able to survive as many as 1000 1-ns pulses at 2 J/cm^2^ (5-Hz repetition rate) before displaying any significant change in its functional performance. These promising results highlight the potential of this class of LC materials in nanosecond-regime, high-peak-power lasers such as OMEGA for applications as polarization control and polarization-smoothing optics. Another distinct advantage of LC optics is that in the event they do sustain damage, they can be refurbished and reinstalled in the laser system with a relatively low cost of materials and effort. The results also illustrate the necessity of taking the molecular structure and electron delocalization of LC mesogens into account when designing new materials for such emerging applications.

For circular polarizer/isolator devices containing the unsaturated chiral dopant CB-15, the 1053-nm, 1.4-ns 1-on-1 LIDT ranged from 17.7 to 30.5 J/cm^2^ depending on input polarization handedness and ellipticity. In 351-nm, multipulse LIFT testing, LC mixtures with an unsaturated chiral-dopant concentration as small as 1 wt% produce nearly immediate and gradual functional degradation under multipulse exposure at 1.8 J/cm^2^ and 5-Hz repetition rate, culminating in a large transmission change onset near ~ 85 pulses. This behavior is attributed to consumption of unsaturated chiral dopant (CB-15) by one or more UV-induced photolytic processes that result in a lengthening of the mixture’s helical pitch (*p*). This finding highlights the need to employ fully saturated –chiral dopants for LC mixtures intended for polarization control applications in the UV region.

## Data Availability

Data underlying the results presented in this paper are not available publicly at this time but may be obtained from the authors upon request.
